# *Burkholderia pseudomallei* musculoskeletal infections (melioidosis) in India

**DOI:** 10.4103/0019-5413.61829

**Published:** 2010

**Authors:** Vivek Pandey, Sripathi P Rao, Sugandhi Rao, Kiran KV Acharya, Sarabjeet Singh Chhabra

**Affiliations:** Department of Orthopadics, Kasturba Medical College, Manipal, Karnataka - 576 104, India; 1 Department of Microbiology, Kasturba Medical College, Manipal, Karnataka - 576 104, India

**Keywords:** *Burkholderia pseudomallei*, melioidosis, musculoskeletal infection

## Abstract

Melioidosis, an infection due to gram negative *Burkholderia pseudomallei*, is an important cause of sepsis in east Asia especially Thailand and northern Australia. It usually causes abscesses in lung, liver, spleen, skeletal muscle and parotids especially in patients with diabetes, chronic renal failure and thalassemia. Musculoskeletal melioidosis is not common in India even though sporadic cases have been reported mostly involving soft tissues. During a two-year-period, we had five patients with musculoskeletal melioidosis. All patients presented with multifocal osteomyelitis, recurrent osteomyelitis or septic arthritis. One patient died early because of septicemia and multi-organ failure. All patients were diagnosed on the basis of positive pus culture. All patients were treated by surgical debridement followed by a combination of antibiotics; (ceftazidime, amoxy-clavulanic acid, co-trimoxazole and doxycycline) for six months except for one who died due to fulminant septicemia. All other patients recovered completely with no recurrences. With increasing awareness and better diagnostic facilities, probably musculoskeletal melioidosis will be increasingly diagnosed in future.

## INTRODUCTION

Melioidosis is an infectious disease, caused by a gram negative obligatory aerobic non-spore forming bacillus, *Burkholderia pseudomallei*.^1^ It was first diagnosed by Captain Alfred Whitmore, and his assistant, C.S. Krishnaswami in 1911 in Burma.^2^ It is a soil saprophyte, present in stagnant water, paddy fields and infection is via the skin through abrasions or inhalation.^3^ Patients with diabetes mellitus, chronic renal failure, alcoholism, cirrhosis and immunocompromised status are more susceptible.^4^,^5^ However, there seems to be no association with human immunodeficiency virus (HIV). It may present at any age though most commonly in the third and fourth decade. The presentation is mostly as abscesses in lung, liver, spleen, parotids and skeletal muscles. It can be localized or disseminated (with or without septicemia).^6^,^7^ Musculoskeletal infection due to melioidosis is not common in India. However, several cases of soft tissue infection have been reported in the past.^8^–^10^ Clinically, it mimics pyogenic bacterial infection, gram negative sepsis, tuberculosis or even polyarthritis.^11^–^13^ An abscess may heal after incision and drainage but may recur. Patient may present with florid pneumonia and rapidly progress to fulminant septicemia with abscess, osteomyelitis or septic arthritis. The diagnosis is likely to be missed by the clinician and microbiologist unless a high degree of suspicion is maintained. Histopathology may show necrotizing granuloma without acid fast bacilli, which may confuse the picture with tuberculosis.^14^ It is sensitive to ceftazidime, amoxy-clavulanic acid, co-trimoxazole and doxycycline and resistant to aminoglycosides, macrolides, second generation cephalosporins, fluoroquinolones and rifamycins.

Many cases of soft tissue or non-musculoskeletal melioidosis have been reported by various authors in the past from India but only recently have more cases of musculoskeletal melioidosis been seen.^8^–^10^ We are reporting five cases of musculoskeletal melioidosis for the first time from India, presenting to our department from September 2005 to December 2007. All patients were followed up for minimum of 12 months. One patient died and there were no recurrences in the rest.

## CASE REPORTS

### Case 1

**Figure 1:**
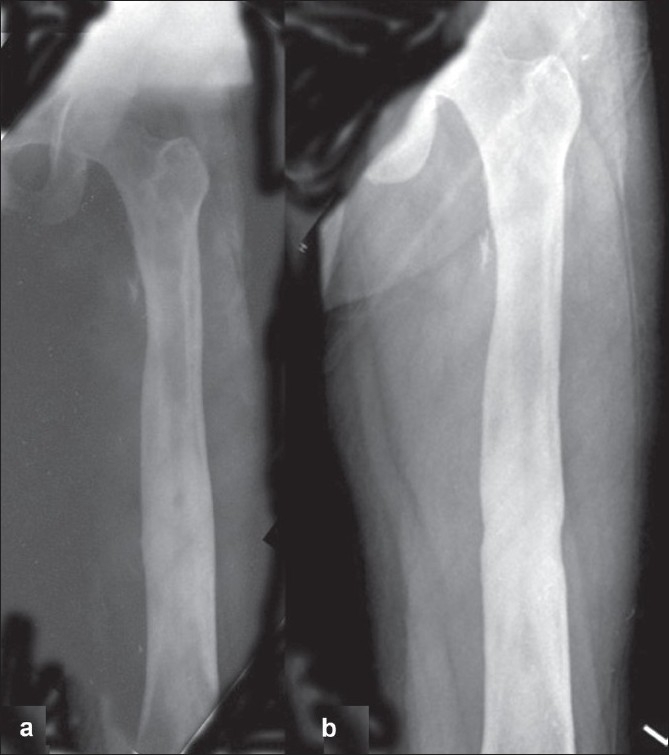
X-ray of left femur anteroposterior view showing (a) femoral cortical thickening. (b) Healed lesion after 30 months

**Figure 2:**
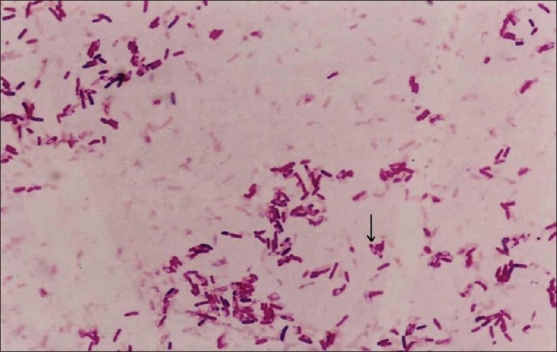
Gram stain picture showing black arrow pointing a bipolar stained *B. pseudomallei*

A 29-year-old male shopkeeper presented with left thigh pain of one-year duration with intermittent fever. Clinically, there was local rise in temperature at mid-thigh with no skin changes or sinuses. Mid-shaft of left femur was thick and tender. Rotational range of motion at hip was full but flexion (0-100°) and abduction (0-30°) were limited. Knee range of motion was 5-100°. His hemoglobin was 12.3 gm% and erythrocyte sedimentation rate (ESR) was 64 mm/h. His serology was negative for HIV. He was nondiabetic. X-ray revealed thickening of mid-shaft femoral cortex [Figure 1a]. Computed tomography scan (CT scan), magnetic resonance imaging (MRI) and bone scan were not asked as the clinical and radiological evidence pointed towards localized osteomyelitis. He underwent curettage and decompression of the femur. Biopsy of the lesion was reported as chronic non-tubercular granulomatous osteomyelitis or Garre's osteomyelitis as differential diagnosis. The local culture was negative for any bacterial, tubercular or fungal growth. Patient was kept on intravenous (IV) cefotaxime and later followed by oral cefuroxime for four weeks. His symptoms reappeared after six months. He underwent repeat curettage and this time, the culture from local site was positive for *Burkholderia pseudomallei*. Gram staining revealed typical bipolar stained *B. pseudomallei* organisms [Figure 2]. After initial three weeks of I.V. ciprofloxacin and amoxy-clavulanic acid, according sensitivity, he was kept on co-trimoxazole for six months with complete clinical and biochemical parameter recovery. His thigh pain and swelling subsided. Range of movement at knee and hip returned to full range and painless. His ESR at final follow-up was 16 mm/h. He has been asymptomatic for 30 months. His final X-ray reveals healed lesion [Figure 1b].

### Case 2

**Figure 3:**
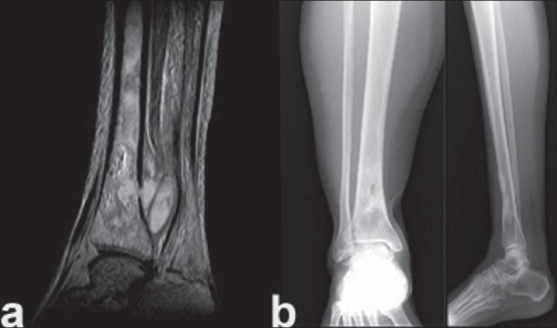
T2W MR image of tibia showing (a) osteomyelitis of tibia with abscess in soft tissue (b) X-ray anterposterior and lateral view of tibia showing healed osteomyelitis of lower end tibia after 28 months

A 40-year-old female, beautician, initially presented to the department of internal medicine with multiple joint pains in knee, ankle and foot with intermittent fever for six weeks. On clinical examination, she had hepato-splenomegaly with low grade fever. Her ESR was 110 mm/h. Her serum titres for salmonella were high. Blood culture was negative. Based on such clinical presentation, she was suspected to have enteric fever and was started on I.V. ceftriaxone. However, there was no clinical response. Later, she developed severe pain in left knee and leg with impending abscess in leg. Sonographic examination of liver and spleen did not show any lesion.Bone scan showed increased uptake and MRI revealed intramedullary abscess of left tibia [Figure 3a]. Decompression and curettage of left leg abcess was performed. Wound was left open to heal with regular dressings. Local pus culture was positive for *B. pseudomallei*. Patient was started on I.V. ceftazidime and amoxy-clavulanic acid. She became afebrile in two weeks time and was started on oral co-trimoxazole and doxycycline at fourth week. The wound healed in six weeks. Six weeks later, she presented with an abscess around ankle while still on antibiotics. She again underwent drainage of abscess and decompression. Again, *B. pseudomallei* was the causative organism. She was re-started on I.V. ceftazidime and amoxy-clavulanic acid for three weeks followed by oral co-trimoxazole and doxycycline for six months. She has remained asymptomatic for 28 months. Her final X-ray reveals healed lesion [Figure 3b].

### Case 3

**Figure 4:**
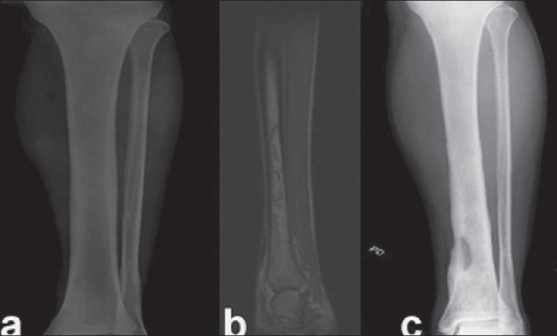
(a) X-ray anteroposterior view of left tibia showing normal appearance. (b) T2W MR image of left tibia showing intramedullary abscess. (c) X-ray anteroposterior view of left tibia shows healed osteomyelitis of lower end tibia after 22 months

A 33-year-old male serviceman was brought to hospital with symptoms of acute left lower lobe pneumonia of one-week duration. CT scan of chest showed segmental consolidation. As he refused admission, he was sent home on oral amoxy-clavulanic acid. He stopped medication within two days. After a week, he came back with severe breathing difficulty, fever and vomiting. His oxygen saturation (SpO_2_) was 76% on room air. He was detected to have type 2 diabetes mellitus. He was admitted with a diagnosis of acute respiratory distress syndrome. His blood culture and sputum culture were negative for bacterial growth. Acid fast bacillus staining was negative for tuberculosis. Bronchial lavage and biopsy were not done as he presented with acute respiratory symptoms. Sputum was not sent for tuberculosis for the same reason as he was diagnosed with acute respiratory distress syndrome and never had any past history of tuberculosis. CT scan of thorax was suggestive of pulmonary infarction. He was managed on I.V. amoxy-clavulanic acid for five days followed by same antibiotic orally and discharged after 10 days. After two days of discharge, he was re-admitted with pain in left tibia and fever. Movements at knee and ankle were normal. He had stopped taking antibiotics after he reached home. He was not immunocompromised. At the time of re-admission, he was on oral amoxy-clavulanic acid. On serological investigations for HIV, he was found to be normal. X-ray of tibia appeared normal [Figure 4a]. However, MRI of tibia showed intramedullary abscess [Figure 4b]. He underwent decompression of tibia. The culture was positive for *B. pseudomallei*. He was started on I.V. ceftazidime and I.V. amoxy-clavulanic acid for three weeks followed by doxycycline and co-trimoxazole for six months. He is asymptomatic for 22 months. His final x-ray reveals healed lesion in lower tibia [Figure 4c].

### Case 4

A 44-year-old male diabetic, policeman, presented with complaints of pain and swelling in left foot for four weeks. Eight weeks prior to presentation, he had drainage of left axillary abscess, which was still draining through a sinus. Clinically, the foot was swollen with two discharging sinuses. There was local rise in temperature with indurated skin. Ankle movements were normal. We explored the foot abscess as well as the axillary sinus. The local culture was positive for *B. pseudomallei*. Patient was started on I.V. ceftazidime and amoxy-clavulanic acid combination for three weeks. Later, he was put on co-trimoxazole and doxycycline for six months. He is asymptomatic for 12 months. His final X-ray revealed healed lesion of tarsal bones.

### Case 5

A 55-year-old diabetic female was brought to the casualty with complaints of fever, dyspnea, left knee pain and swelling. Chest X-ray showed extensive consolidation and left knee aspiration revealed pus. Both blood culture and local pus culture showed *B. pseudomallei*. She remained hypoxic, hypotensive and hyponatremic in spite of all efforts and later succumbed to septicemia.

## DISCUSSION

Melioidosis is a relatively rare infectious disease of musculoskeletal system. It has got widespread occurrence and it is found in soil of almost all the states of India. However, it is reported more from southern states.^8^–^10^ India's rural population lives in close proximity to agricultural land and is quite susceptible to this neglected killer disease. It occurs in our patients with far more frequency than imagined. But it is underreported in India because of: (i) lack of awareness of disease (ii) low index of suspicion and (iii) under-recognition of disease.^14^,^15^ It should be suspected by a microbiologist on isolation of a gram negative bacillus, which is oxidase positive, bipolar staining which is gentamycin resistant.

It mostly affects the respiratory system with soft tissues abscesses. It can be ignored easily by laboratory technicians or clinicians if not aware of it. It occurs in persons with concur rent diabetes, renal failure, thalassemia and immunocompromised status. It is introduced into the body by inoculation via skin or inhalation.^14^ Sexual transmission and vertical transmission at birth is also reported.^16^,^17^ It is not a zoonosis.^18^ The highest concentration of organism was found to be on the surface water of wet rice fields though none of our patients gave any history of recent entry into rice fields or any suspected contaminated area.^18^ The clinical presentation is quite variable. It can mimic conditions from acute or chronic forms of infection to various rheumatoid disorders.^14^ Patient had initial presentation of chronic granulomatous osteomyelitis due to melioidosis, which remains indistinguishable from tuberculosis or staphylococcal abscess except by microbiological culture.^10^,^19^ One should always consider melioidosis as a differential diagnosis with atypical presentations especially if patient is from endemic area. One patient presented initially with polyarthralgia-like symptom whose serology was similar to typhoid. Later, she presented with multifocal osteomyelitis in leg and foot. Three of them were diabetics. In summary, none had similar presentation, which justifies that musculoskeletal melioidosis can mimic common diseases. In musculoskeletal melioidosis, the diagnosis is usually made by culture from the pus. Blood culture is usually negative. Local pus culture was positive in all cases, while blood culture was positive only in the septicemic case. Even though various indirect hemagglutination tests are reported, we have no experience in using it for diagnosis.^20^ *Burkholderia pseudomallei* are sensitive to ceftazidime, amoxy-clavulanic acid, chloramphenicol, tetracycline and co-trimoxazole.^21^,^22^ Imipenem is also quite effective especially in septicemic type.^23^ All our cases were sensitive to these antibiotics. Usually operative intervention along with combination antibiotics (IV ceftazidime and amoxy- clavulanic acid) for 2–3 weeks is instituted instead of a single antibiotic to decrease the chances of recurrence, followed by maintenance oral antibiotic combination therapy of doxycycline, chloramphenicol and co-trimoxazole for 4–6 months. However, we did not use chloramphenicol due to known complication of bone marrow suppression. We used initial combination I.V. antibiotics for three weeks because we felt that this is a slow responsive and difficult condition to treat with known recurrences. It may take about two weeks for fever to subside. Disseminated form may land up in ICUs with increased mortality. We used I.V. ceftazidime and amoxy-clavulanic acid for 2–3 weeks followed by oral therapy with doxycycline and co-trimoxazole for six months. Imipenem is recommended for septicemic form.

Since the organism is resistant to aminoglycosides, macrolides, second generation cephalosporins, rifamycin and fluoroquinolones, hence it is important to start the right antibiotic as any attempt of starting these antibiotics by assumption will certainly lead to failure of therapy.

All our patients had full recovery but for one who died because of disseminated infection, which has high mortality.^2^ Till now, there has been no relapse in any of our patients. However, 10% relapse even after 20 weeks of treatment is reported. However, relapse rate increases to 30% if duration of treatment is less than eight weeks.^24^ We realize that distribution and frequency of musculoskeletal melioidosis is probably greatly underestimated.^25^ It is quite difficult to prevent it in rice producing areas and probably this is why it is more common in southern India. The longer duration of treatment and the cost of antibiotic therapy are important issues. The status of vaccination against infection is poor as repeated natural immunization does not offer any protection. Awareness of this infection, with all its forms of presentation will help early detection, isolation of the organism and disease management.
